# Total Fluid and Water Consumption and the Joint Effect of Exposure to Disinfection By-Products on Risk of Bladder Cancer

**DOI:** 10.1289/ehp.10281

**Published:** 2007-08-29

**Authors:** Dominique S. Michaud, Manolis Kogevinas, Kenneth P. Cantor, Cristina M. Villanueva, Monteserrat Garcia-Closas, Nathaniel Rothman, Nuria Malats, Francisco X. Real, Consol Serra, Reina Garcia-Closas, Adonina Tardon, Alfredo Carrato, Mustafa Dosemeci, Debra T. Silverman

**Affiliations:** 1 Department of Epidemiology, Harvard School of Public Health, Boston, Massachusetts, USA; 2 Channing Laboratory, Department of Medicine, Brigham and Women’s Hospital and Harvard Medical School, Boston, Massachusetts, USA; 3 Division of Cancer Epidemiology and Genetics, National Cancer Institute, National Institutes of Health, Department of Health and Human Services, Bethesda, Maryland, USA; 4 Centre for Research in Environmental Epidemiology, Municipal Institute of Medical Research, Barcelona, Spain; 5 Medical School, University of Crete, Heraklion, Greece; 6 Universitat Pompeu Fabra, Barcelona, Spain; 7 Consorci Hospitalari Parc Taulí, Sabadell, Spain; 8 Unidad de Investigación, Hospital Universitario de Canarias, La Laguna, Spain; 9 Universidad de Oviedo, Oviedo, Spain; 10 Hospital General Universitario de Elche, Elche, Spain

**Keywords:** bladder cancer, case–control study, chlorination by-products, fluid intake, water intake

## Abstract

**Background:**

Findings on water and total fluid intake and bladder cancer are inconsistent; this may, in part, be due to different levels of carcinogens in drinking water. High levels of arsenic and chlorinated by-products in drinking water have been associated with elevated bladder cancer risk in most studies. A pooled analysis based on six case–control studies observed a positive association between tap water and bladder cancer but none for nontap fluid intake, suggesting that contaminants in tap water may be responsible for the excess risk.

**Objectives:**

We examined the association between total fluid and water consumption and bladder cancer risk, as well as the interaction between water intake and trihalomethane (THM) exposure, in a large case–control study in Spain.

**Methods:**

A total of 397 bladder cancer cases and 664 matched controls were available for this analysis. Odds ratios (OR) were estimated using unconditional logistic regression, controlling for potential confounders.

**Results:**

Total fluid intake was associated with a decrease in bladder cancer risk [OR = 0.62; 95% confidence interval (CI), 0.40–0.95 for highest vs. lowest quintile comparison]. A significant inverse association was observed for water intake (for > 1,399 vs. < 400 mL/day, OR = 0.47; 95% CI, 0.33–0.66; *p* for trend < 0.0001), but not for other individual beverages. The inverse association between water intake and bladder cancer persisted within each level of THM exposure; we found no statistical interaction (*p* for interaction = 0.13).

**Conclusion:**

Findings from this study suggest that water intake is inversely associated with bladder cancer risk, regardless of THM exposure level.

Established risk factors for bladder cancer, including smoking and high-risk occupational exposures (Silverman et al. 2006), contain known carcinogenic compounds (e.g., aromatic amines) that may form DNA adducts in the bladder when not excreted promptly. Voiding frequency is a main determinant of DNA adduct formation of known bladder carcinogens in dogs (Kadlubar et al. 1991), and it has been hypothesized that a high fluid intake may reduce bladder cancer risk by increasing urination frequency. In support of this hypothesis, a significantly lower risk of bladder cancer was observed among individuals with high fluid intake in a large prospective study of men (Michaud et al. 1999). Alternatively, high water intake may increase bladder cancer risk if contaminants such as arsenic or chlorinated by-products are elevated in the water source (Villanueva et al. 2007), or through other alternative mechanisms.

Overall, studies on water or total fluid intake and bladder cancer have been inconsistent (Bruemmer et al. 1997; Cantor et al. 1987; Geoffroy-Perez and Cordier 2001; King and Marrett 1996; Koivusalo et al. 1998; McGeehin et al. 1993; Mills et al. 1991; Slattery et al. 1988; Vena et al. 1993; Wilkens et al. 1996; Zeegers et al. 2001). The association between water intake and bladder cancer risk has been complicated by the possibility that water contaminants, especially disinfection by-products and arsenic, may increase the risk of bladder cancer [summarized by Silverman et al. (2006)]. In a recent pooled analysis of six case–control studies with data on fluid intake and chlorination by-products, tap water was associated with an elevated risk of bladder cancer at all levels of trihalomethane (THM) exposure among men but not women (Villanueva et al. 2006b).

We examined the potential role of water and total fluid intake on bladder cancer risk and the effect of exposure to disinfection by-products in water simultaneously in a large multi-center case–control study conducted in Spain.

## Methods

### Study population

Between June 1998 and June 2001, a hospital-based case–control study of bladder cancer was conducted in multiple centers in Spain. Cases and controls were recruited from 18 participating hospitals in five geographic areas of Spain (3 in Barcelona, 2 in Vallès/Bages, 1 in Alicante, 2 in Tenerife, and 10 in Asturias). Cases, defined as histologically confirmed primary bladder cancer patients (urothelial carcinoma), were identified by the urologic services at diagnosis. Bladder cancer cases were eligible if they were residents of the geographic catchment area of the participating hospitals and were between 20 and 80 years of age. Research staff frequently reviewed hospital discharge records and pathology records to ensure that no cases were missed.

Controls were selected from the same hospitals at about the time the case patients were diagnosed (median time between the case interview and the interview of the matched control was 150 days), and were individually matched 1:1 on sex, age at diagnosis/interview (within 5 years), and geographic area of residence. Hospital patients admitted for conditions that could be related to exposures under investigation, such as smoking, were not selected as controls for this study. Controls were admitted for the following reasons: hernias (33%), other abdominal surgery (12%), fractures (24%), other orthopedic problems (6%), hydrocele (13%), circulatory disorders (5%), dermatologic disorders (2%), ophthalmologic disorders (2%), and other diseases (3%). The study was approved by the human subjects review board of each participating institution, and all participants provided signed informed consent before being enrolled in the study.

### Interview data

During hospitalization, cases and controls were interviewed by trained interviewers, who used computer-assisted software to record the information directly during the interview. Information collected during the interview included sociodemographic characteristics, family history of cancer, smoking history, occupational history, residential history (all residences of at least 1 year beginning at birth), drinking water source at each residence (municipal/bottled/private well/other), and medical history. Of the 1,457 eligible cases, 84% were interviewed (*n* = 1,219); 87% (*n* = 1,271) of the 1,465 eligible controls were interviewed. For the in-person interview, cases and controls were instructed to report usual adult lifetime consumption of beverages.

In addition, cases and controls were instructed on how to complete a food-frequency questionnaire (FFQ), which was then self-administered. For the FFQ, cases and controls were asked to report diet intake during the past 5 years. Specific beverage consumption, including water, was added to the interview after the study had been in the field for almost 1 year because of concerns that the FFQ might not provide sufficiently detailed data. For each beverage, a serving size was specified, and categories of intake frequency were provided. Intake of total fluid was estimated by multiplying volume and frequency of intake and summing overall individual beverages for both the personal interview and the FFQ. Questions on beverages included coffee, beer, wine, liquor, champagne, soda, juices, tea, milk, and water.

### Historical THMs

The method for calculating THMs is described in detail elsewhere (Villanueva et al. 2006a). Briefly, annual average THM levels, water source history since 1920, and the year that chlorination began were obtained from local authorities and water companies; this information was available for 78.5% of the total study person-years. Individual and municipal databases were merged by year and municipality to obtain individual year-by-year average THM levels. Residential THM exposure (micrograms per liter) was based on the time-weighted annual average municipal THM level at all residences since the age of 15 years (years prior to age 15 were excluded to minimize missing or poorly recalled residential data in those early years). We used the same cutpoints as those used in the main study on THM levels (Villanueva et al. 2007), and these were based on the distribution of controls. We derived other THM variables for the present study, but the association with risk of bladder cancer was strongest for the residential THM variable as reported by Villanueva et al. (2007) and was thus selected for this analysis. We assumed that the residential THM exposure was zero if drinking water came from a private well, bottled water, or another nonmunicipal source, or if the study period was before municipal chlorination started.

### Statistical analysis

We excluded from our analyses individuals with missing interview data on fluids (*n* = 1,412; questions on fluid intake were added to in-person interviews after study initiation), missing smoking data (*n* = 12), non-white subjects (*n* = 1), unsatisfactory overall quality of interview (*n* = 1), and subjects with nonurothelial carcinoma (*n* = 3). The final data set for this analysis consists of 1,061 individuals (397 cases and 664 controls).

To estimate the relation between our exposures of interest and bladder cancer risk, we calculated odds ratios (ORs) and 95% confidence intervals (CIs) using unconditional logistic regression models, adjusting for matching factors (age at diagnosis/interview, geographic region, sex) and other potential confounding variables (smoking status/duration, nighttime urination frequency, and exposure to THMs). Unconditional models were used to increase statistical power by inclusion of unmatched pairs. In most models, and unless otherwise specified, we included only smoking status in the models because including categories for smoking duration did not change the estimate of interest. In addition, high-risk occupations (painters, paper-hangers, and plasterers; truck drivers and tractor-trailer drivers; railroad brake, signal, and switch operators; sailors and deckhands; precision laundering, cleaning, and dyeing occupations; textile machine setup operators; welders and solderers; general building contractors; heavy construction workers; yarn and thread mill workers; and textile workers) were also included in the final models. Quintiles for total fluid intake were created based on the distribution among controls. For most individual beverages, nondrinkers formed one category that we used as the reference group, and the remaining individuals were grouped into categories based on the distribution among controls and range of available data for each item. Tests for trend were conducted using the median value for each level of the categorical variable among controls and entering this variable as a continuous variable in the models.

We examined interaction between water intake volume and THM levels by creating cross-product terms of continuous variables and including them in the logistic regression models. For these analyses, we included only individuals who had complete fluid data from the interview and for whom information on residential THM history was ≥ 70% (292 cases and 487 controls).

## Results

In this subpopulation, cases and controls were similar with respect to age, sex, geographic area, and education levels, but cases were more likely to be current smokers than controls (Table 1). Mean and median water intake was similar by admission diagnosis of control patients (means ranged between 844 and 896 mL/day, and the median was 700 mL/day in each of the diagnostic categories that contributed to > 2% of total controls). Among male controls, water made up the majority of fluids (42%), followed by milk (14%), wine (14%), coffee (8%), beer (6%), juice (4%), soda (4%), and other beverages (8%). Female controls consumed substantially less alcohol than males; their fluid consumption was as follows: water (50%), milk (23%), coffee (12%), juice (5%), wine (3%), and other beverages (7%).

A statistically significant 39% decrease in the risk of bladder cancer was observed for males and females in the highest versus lowest levels of total fluid intake (Table 2), but the test for trend did not reach statistical significance (*p* for trend = 0.07). Unadjusted ORs were slightly attenuated compared with those in the fully adjusted model (Table 2). Exclusion of THM level from the models had little or no impact on estimates of risk (data not shown). The associations were similar in males (OR = 0.61; 95% CI, 0.38–0.97) and females (OR = 0.58; 95% CI, 0.18–1.87) for the same comparisons of highest versus lowest intake of total fluid. For water intake, we observed a 53% decrease in risk for males and females consuming ≥ 1,400 mL/day compared with those consuming < 400 mL/day. The associations were similar when stratified on sex comparing > 1,399 mL/day with < 400 mL/day: for males OR = 0.47 (95% CI, 0.33–0.68) for females OR = 0.61 (95% CI, 0.23–1.65). Risk decreased with increasing water intake among never, past, and current smokers, although only the trend among past smokers was statistically significant (Table 3). We did not observe a statistically significant interaction between smoking status and water intake (*p* for interaction = 0.32); never, past, and current smokers had a 51%, 67%, and 44% lower risk of bladder cancer, respectively, for > 1,399 mL/day vs. < 400 mL/day.

We examined the joint effect of water intake and THM exposure by creating four categories of residential THM exposure, as reported previously by Villanueva et al. (2007). The estimates for THM levels by quartiles in this data set were similar to those previously published (Table 4). We observed a greater than 2-fold increase in risk of bladder cancer among individuals with elevated residential THM levels who consumed ≤ 400 mL/day of water, compared with those consuming the same amount of water but with low THM levels (Table 4). In the low THM strata, high versus low water intake was associated with a significantly lower risk of bladder cancer (OR = 0.36; 95% CI, 0.15–0.83). In the highest THM strata, high versus low water intake was also associated with a lowering of risk. However, we found no interaction between THM exposure and water intake (*p-*interaction = 0.13).

Consumption of individual beverages other than water, including coffee, beer, and wine, was not related to risk of bladder cancer in multivariate models (OR for highest vs. lowest category of beverage intake ranged between 0.97 and 1.17). Similarly, total fluid intake not including water was not associated with risk (OR = 0.84; 95% CI, 0.53–1.33, for highest vs. lowest quintile comparison, controlling for water in the model).

## Discussion

In this case–control study conducted in Spain, we observed an inverse association for total fluid intake that was mostly driven by water intake. A 53% lower risk of bladder cancer was observed in individuals who consumed ≥ 1,400 mL of water per day compared with those who consumed < 400 mL/day after adjusting for known and potential confounders. The inverse association for water intake was present across all strata of smoking status. Similarly, higher water intake was associated with lower bladder cancer risk within each THM exposure strata.

The inverse association for water intake and bladder cancer risk in the present study is consistent with findings from a prospective cohort (Michaud et al. 1999). In contrast to that study, however, we did not observe inverse associations for other beverage items combined. Given that the main biological hypothesis is that fluids “flush” out carcinogens, or reduce their contact time with the urothelium (Kadlubar et al. 1991), it is unclear why other beverages that also contribute fluid volume are not inversely associated with risk. One reason for this observation may be that water consumption in this population better reflects long-term intake, if consumption is consistent over time; in contrast, consumption of other beverages, such as soft drinks, may be more prone to change over time.

The fluid results based on the FFQ were similar to those obtained using the in-person interviews among individuals who completed both an in-person interview and an FFQ (data not shown). For females, results were also identical when comparing data for all subjects who responded to the FFQ to the subset with fluid data obtained during the in-person interview. For males, the water results were slightly attenuated using all subjects who answered the FFQ compared with results from the FFQ on those with in-person interviews only (OR = 0.77; 95% CI, 0.56–1.07, comparing > 1,399 mL/day with < 400 mL/day). This difference could be caused by measurement error in the overall FFQ responders, because those who completed the FFQ and did not have in-person water data were more like to have FFQ errors (defined as double entries or blank items; 58% any error vs. no errors) than those who completed both an FFQ and an in-person interview (48% any error vs. no errors). These data suggest that the FFQ data in this population may have been more prone to error than the in-person interview data.

The inconsistencies in findings on fluid/water intake and bladder cancer risk are apparent in both cohort and case–control studies. The Netherlands Cohort Study (Zeegers et al. 2001) did not replicate the inverse association for total fluid intake and bladder cancer that was observed in the Health Professionals Follow-up Study (Michaud et al. 1999). In one meta-analysis on fluid intake and bladder cancer, Zeegers et al. (2004) concluded that “there is possible evidence that total fluid intake is not associated with bladder cancer.” In contrast, in a pooled analysis of six case–control studies (2,729 bladder cancer cases), Villanueva et al. (2006b) reported that total fluid intake was associated with an increased risk of bladder cancer in men, but not women (the present study was not part of this pooled analysis). Inconsistencies may be caused by differences in exposures to disinfection by-products and other water contaminants that can vary substantially by study population. In the pooled study by Villanueva et al. (2006b), tap water intake was associated with increased risk of bladder cancer at the lowest THM exposure level, and bladder cancer risk increased within each category of THM exposure level. In contrast, in the present analysis, higher water intake reduced the risk of bladder cancer, even among those exposed to the highest levels of THM. It is possible that water contaminants other than disinfection by-products were present in one or more of the pooled studies, thereby leading to different results.

Other methodologic issues may also have contributed to positive findings for total intake or water intake in previous studies. Past studies have varied substantially in how fluid intake was assessed; differences such as the number of questions related to fluid consumption and the period of reference used for beverage intake (e.g., lifetime average intake, intake for period 2 years before interview/diagnosis, adulthood exposure) could have contributed to inconsistencies in findings. For example, fluid intake in years close to diagnosis may not be the relevant exposure, given the long latency for bladder cancer. Changes in fluid intake occurring in patients before diagnosis may also have introduced bias.

The strengths of the present study include detailed interview data on consumption of individual beverages in usual adult intake, detailed assessment of THM exposure, detailed smoking data to adjust for confounding, and high response rates.

As with any case–control study, recall bias is a concern in drawing inferences because it is possible that some differential reporting of water intake may have occurred between cases and controls. However, recall bias tends to occur when cancer patients attempt to find an explanation for their condition and consequently overreport consumption of a “bad” exposure, rather than vice versa, as in this situation where water appears to be beneficial. Furthermore, in the Spanish population there is no general perception of a beneficial effect of high water intake (whereas it is likely to be the case in the United States). Even though selection bias could have occurred in this study because the controls were selected from hospitals, we saw no differences in water intake by control diagnostic category. We also observed very similar associations for smoking (Samanic et al. 2006) and THM levels (Villanueva et al. 2007) as reported previously for this population, suggesting that the present study is representative of the overall study population.

In summary, results from the present study suggest that water intake is inversely associated with the risk of bladder cancer. The decrease in bladder cancer risk observed with higher water intake was perceivable among current, past, and never smokers and for low and high THM exposures alike.

## Figures and Tables

**Table 1:** Characteristics of the participants with complete beverage data in a case–control study in Spain, 1998–2001.

	Cases (*n* = 397) [No. (%)]	Control (*n* = 664) [No. (%)]
Sex		
Male	338 (85.1)	582 (87.7)
Female	59 (14.9)	82 (12.3)
Age (years)		
< 40	10 (2.5)	8 (1.2)
40–44	13 (3.3)	20 (3)
45–49	14 (3.5)	25 (3.8)
50–54	36 (9.1)	55 (8.3)
55–59	32 (8.1)	76 (11.4)
60–64	58 (14.6)	99 (14.9)
65–69	89 (22.4)	167 (25.1)
70–74	73 (18.4)	120 (18.1)
≥ 75	72 (18.1)	94 (14.2)
Geographic area		
Barcelona	75 (18.9)	136 (20.5)
Valles	54 (13.6)	93 (14)
Alicante	37 (9.3)	41 (6.2)
Tenerife	84 (21.2)	138 (20.8)
Asturias	147 (37)	256 (38.5)
Smoking status		
Never	79 (19.9)	240 (36.1)
Former	144 (36.3)	247 (37.2)
Current	167 (42.1)	153 (23)
Occasional	7 (1.8)	24 (3.6)
Education		
< Primary school	174 (43.8)	320 (48.2)
< High school	163 (41.1)	243 (36.6)
≥ High school	57 (14.4)	97 (14.6)
Other	3 (0.7)	4 (0.6)
High-risk occupation^a^		
No	310 (78.1)	574 (86.5)
Yes	74 (18.6)	67 (10.1)
Missing	13 (3.3)	23 (3.5)

a Occupations defined in “Methods.”

**Table 2:** Total fluid and water intake and bladder cancer risk.

	Cases/controls	OR^a^	OR^b^	95% CI^b^	*p-*Trend
Total fluid quintiles^c^					
1	98/134	1.0	1.0	Reference	
2	76/132	0.80	0.67	0.44–1.02	
3	81/134	0.82	0.72	0.48–1.09	
4	73/132	0.76	0.68	0.45–1.04	
5	69/132	0.72	0.62	0.40–0.95	0.05
Water intake (mL/day)					
< 400	155/190	1.0	1.0	Reference	
400–1,399	144/237	0.75	0.71	0.51–0.98	
> 1,399	98/237	0.51	0.47	0.33–0.66	< 0.0001

a Adjusting only for age and sex.

b Adjusting for age, sex, region, cigarette smoking, high-risk occupation, nighttime urination frequency, THM levels, and nontap fluid for water intake.

c Quintiles are sex-specific as follow for males, < 1,375, 1,375–1,800, 1800.1–2,249, 2249.1–2825.5, and > 2825.5 mL/day; for females, < 1,129, 1,129–1,400, 1400.1–1682.5, 1682.6–2259.5, and > 2259.5 mL/day.

**Table 3:** Water intake and bladder cancer risk stratified by smoking status.

	Never-smoker			Past smoker			Current smoker		
Water intake (mL/day)	Cases/controls	OR^a^	95% CI	Cases/controls	OR^a^	95% CI	Cases/controls	OR^a^	95% CI
< 400	29/75	1.0	Reference	63/54	1.0	Reference	61/54	1.0	Reference
400–1,399	32/88	0.95	(0.49–1.85)	47/92	0.42	(0.24–0.76)	62/48	0.99	(0.56–1.75)
> 1,399	18/77	0.49	(0.23–1.05)	34/101	0.33	(0.18–0.59)	44/51	0.56	(0.30–1.05)
*p* for trend		0.05			< 0.001			0.06	

a Adjusting for age, sex, region, high-risk occupation, THM level, nighttime urination frequency, nontap fluid intake, and smoking duration for past and current smokers.

**Table 4:** Joint effect of water and THM levels on bladder cancer risk.

	THM level											
	≤8 μg/L			8–26 μg/L			26–49 μg/L			> 49 μg/L		
Water intake (mL/day)	Cases/controls	OR^a^	95% CI	Cases/controls	OR^a^	95% CI	Cases/controls	OR^a^	95% CI	Cases/controls	OR^a^	95% CI
< 400	25/34	1.0	Reference	21/37	0.92	(0.37–2.28)	43/36	2.63	(1.05–6.55)	29/28	2.07	(0.68–6.28)
400–1,399	30/50	0.66	(0.31–1.41)	22/51	0.69	(0.29–1.63)	25/34	1.08	(0.41–2.83)	27/44	1.16	(0.38–3.54)
> 1,399	16/46	0.36	(0.15–0.83)	8/29	0.40	(0.14–1.17)	22/39	1.07	(0.40–2.88)	24/59	0.80	(0.26–2.51)
Overall	71/130	1.0	Reference	51/117	1.02	(0.54–1.90)	90/109	2.34	(1.16–4.71)	80/131	2.06	(0.83–5.08)

a Adjusting for age, sex, region, cigarette smoking, high-risk occupation, nighttime urination frequency, and nontap fluid intake.
